# Intestinal microbiome–rheumatoid arthritis crosstalk: The therapeutic role of probiotics

**DOI:** 10.3389/fmicb.2022.996031

**Published:** 2022-10-18

**Authors:** Yeboah Kwaku Opoku, Kwame Kumi Asare, George Ghartey-Quansah, Justice Afrifa, Felicity Bentsi-Enchill, Eric Gyamerah Ofori, Charles Kwesi Koomson, Rosemary Kumi-Manu

**Affiliations:** ^1^Department of Biology Education, Faculty of Science Education, University of Education, Winneba, Ghana; ^2^Department of Biomedical Sciences, School of Allied Health Sciences, College of Allied Health Sciences, University of Cape Coast, Cape Coast, Ghana; ^3^Department of Medical Laboratory Science, University of Cape Coast, Cape Coast, Ghana; ^4^Department of Integrated Science Education, Faculty of Science Education, University of Education, Winneba, Ghana

**Keywords:** rheumatoid arthritis, gut microbiome, probiotics, dysbiosis, therapy, inflammation

## Abstract

Rheumatoid arthritis (RA) is a common systemic autoimmune disease with a global health importance. It is characterized by long-term complications, progressive disability and high mortality tied to increased social-economic pressures. RA has an inflammatory microenvironment as one of the major underlying factors together with other complex processes. Although mechanisms underlying the triggering of RA remain partially elusive, microbiota interactions have been implicated. Again, significant alterations in the gut microbiome of RA patients compared to healthy individuals have intimated a chronic inflammatory response due to gut dysbiosis. Against this backdrop, myriads of studies have hinted at the prospective therapeutic role of probiotics as an adjuvant for the management of RA in the quest to correct this dysbiosis. In this article, the major gut microbiome alterations associated with RA are discussed. Subsequently, the role of the gut microbiome dysbiosis in the initiation and progression of RA is highlighted. Lastly, the effect and mechanism of action of probiotics in the amelioration of symptoms and severity of RA are also espoused. Although strain-specific, probiotic supplementation as adjuvant therapy for the management of RA is very promising and warrants more research.

## Introduction

Rheumatoid arthritis (RA) is a common systemic autoimmune disease with a global prevalence of about 0.5–1%. It is characterized by long-term complications, progressive disability and high mortality tied to increased social-economic pressures (Firestein, 2003; Mikuls, 2003). Irrespective of the devastating effect of RA on populations, its etiology remains elusive. However, environmental and genetic effects have been intimated as the major culprits. These culminate in the pathophysiological destruction of joints due to the inability of the immune system to distinguish self-antigens of the joint and its associated tissues from non-self, this initiating a cascade of inflammatory reactions (Opoku et al., 2020). The observed inflammatory cascade results in articular damage mainly driven by lymphocytes, macrophages and fibroblast-like synoviocytes (FLS; McInnes et al., 2011). These underlying reactions lead to pathological changes which result in the destruction of cartilage and subchondral bone, infiltration of inflammatory cells and synovitis with associated hyperplasia (Gu et al., 2014).

Like many other chronic diseases and their sequelae, RA has an inflammatory microenvironment as one of the major underlying factors (Opoku et al., 2020). Although inflammation is part of the innate immune system and plays a critical role in the removal of pathogens, damaged cells and irritants, it is only advantageous when short-termed and under control (acute inflammation). However, when prolonged than necessary, known as chronic inflammation, it becomes disadvantageous and contributes to concurrent obliteration and curing of tissues (Aggarwal et al., 2009).

Although mechanisms underlying the triggering of RA remain partially elusive, microbiota interactions have been implicated (Ruff and Kriegel, 2015; Corrêa et al., 2019). These microbiome alterations have been reported especially in the gut and oral microbiome. Again, these alterations or changes in the microbiome have been recently reported as key environmental risk factors in the establishment of RA (Scher et al., 2016). It is highly believed that the neonatal period is critical for the maturation of T and B cells and the development of lymphoid structures tied to the acquisition of immune tolerance to gut commensals (Zeissig and Blumberg, 2014; Zhao and Elson, 2018). Research has intimated the possibility of dysregulation and subsequent immune-mediated diseases such as autoimmunity and allergies when microbial communities are altered during this time of development (Dehner et al., 2019). Bacteroidetes and Firmicutes are the two major phyla which are known to dominate the microbiota of humans even though their abundance in fecal samples remains relatively constant in healthy individuals.

### Microbiome changes in RA

Humans from inception have constantly coexisted and coevolved with trillions of microbes colonizing most body surfaces and cavities. This is normally referred to as the human microbiome. The gastrointestinal tract harbors most of the bacteria present in the body with most of these organisms from the phyla Bacteroidetes and Firmicutes (Li and Wang, 2021). Together with other organisms from different phyla such as Cyanobacteria, Actinobacteria, Proteobacteria, Fusobacteria, Verrucomicrobia etc., they play key roles in the establishment and maintenance of homeostasis in the immune system (De Santis et al., 2015).

Although this microbiome can remain resilient through the life of an individual, its composition is liable to alterations by factors such as age, sex, genes, drugs, diet, diseases, etc. (Clemente et al., 2018). Previously difficult to study, the advent of modern technologies such as shotgun sequencing has made it possible to study the possible constitution and alteration of the microbiome.

Several animal model experiments gave earlier intimation of the role of gut microbiota in the initiation and progression of RA (Taurog et al., 1994; Abdollahi-Roodsaz et al., 2008; Wu et al., 2010). In these studies, it was observed that most of these susceptible animal models of RA under germ-free conditions remained healthy only to develop RA after exposure to certain microbes. Yamamoto et al. (2000), hinted at the role of the gut microbiome in arthritis when they reported a chronic animal model of polyarthritis by hyper-immunization with attenuated *Enterococcus faecalis*. In a related study by Dorożyńska et al. (2014), escalated arthritic symptoms were reported in patients with partial depletion of their gut microbiome induced by antibiotics. Other studies have reported a significant decrease in *Haemophilus* spp. tied to an upsurge in the levels of *Lactobacillus salivarius* in saliva, gut and dental microbiome of patients with RA compared to their healthy counterparts. Interestingly, this alteration or dysregulation was partly restored after treatment with Disease-modifying antirheumatic drugs (DMARDs; Zhang et al., 2015). Recently, a study has reported a significant decrease in *Lactobacillus*, *Enterobacter*, *Alloprevotella* and *Odoribacter* coupled with an upsurge in the number of genera *Escherichia–Shigella* and *Bacteroides* in some Chinese patients with RA. In a similar Japanese cohort of RA patients, however, there was an increase in the abundance of the genus *Prevotella* (Sun et al., 2019). Again, anticitrullinated peptide antibody (ACPA)-positive RA patients were associated with an increased amount of *Clostridiales*, *Blautia* and *Akkermansia* compared to ACPA-negative RA individuals (Chiang et al., 2019). Also, other specific oral bacteria responsible for periodontal diseases such as *Prevotella intermedia*, *Aggregatibacter actinomycetemcomitans* and *P. gingivalis* have all been associated with the initiation of RA (Mei et al., 2021). In a current related study, the genus *Bifidobacterium* from the phylum Actinobacteria and genus *Dialister* reported significant increases in patients with RA compared to healthy controls (Wang et al., 2022).

To further buttress the alteration in the gut microbiome, Mei et al. (2021), associated a rich composition of *Neisseria* spp., *Streptococcus* spp. and *Haemophilus* spp. with RA patients. However, in the same patients, significant depletion of other species such as *Fusobacterium varium*, *Clostridium celatum* and *Enterococcus faecalis* were recorded. A careful analysis of these species clearly shows that species enriched under RA conditions belonged to *Proteobacteria* and *Firmicutes* while healthy controls are enriched with species from the *Firmicutes* genera. Again, *Enterococcus faecalis* have been observed in both synovial tissues and blood cultures of patients with RA (Luo et al., 2017; Chandradevan et al., 2020).

Again, evidence from both clinical and experimental studies has suggested alterations in the gut microbiome during the treatment of patients with RA (Mei et al., 2021). In one study, etanercept, a tumor necrosis factor-alpha (TNF-α) inhibitor, made significant alterations and partly improved the microbiota in patients with RA (Picchianti-Diamanti et al., 2018). Similarly, in animal studies involving collagen-induced arthritic mice, etanercept significantly reduced *Escherichia/Shigella* while increasing the composition of *Tannerella*, *Lactobacillus* and *Clostridium XIVa* (Wang et al., 2020). Furthermore, other natural compounds which have demonstrated enormous prospects for the alleviation of arthritis-associated dysbiosis in gut microbiome composition such as Clematis triterpenoid saponins have also proved effective in the improvement of arthritis symptoms (Guo et al., 2019). Recently, the emergence of data on microbiomes associated with different treatments has provided new insights regarding the associations among RA treatments, the gut microbiota, and clinical outcomes.

### Role of the microbiome in RA

The gut microbiota play a critical role in the complexity and dynamics of host immune homeostasis (D’Amelio et al., 2018). According to Wu et al. (2016), the immune system of the mucosa is involved in the development and maintenance of a healthy microbiome in the gut. The gut microbiome alters colonic regulatory T cells (Tregs) to maintain the immune homeostasis and functions (Tanoue et al., 2016). Tregs play a vital role in the immune system by secreting anti-inflammatory cytokines to aid in the suppression of unwarranted activation of effector T cells (Kayama et al., 2020). The interdependence and relationship of the gut microbiome and dysbiosis influence individual susceptibility to immune-mediated diseases such as type 1 diabetes, RA etc. (de Oliveira et al., 2017). This is possible as disruption of immune response due to dysbiosis of gut microbiome is often not localized but rather can affect other unrelated anatomical sites some distance away (Cho and Blaser, 2012).

In the development of RA, different species of microbes in the gut microbiota may either play a protective or a pathogenic role (Mizuno et al., 2017). Species of Prevotella such as *P. copri* are suggested to influence the initiation and progression of RA. This is made evident as microbes such as *P. copri* highly stimulates the upregulation of Th17-related cytokines including IL-6 and IL-23 tied to the elevated responsiveness of Treg cells to dendritic cells (Maeda et al., 2016). This leads to an increase in the recruitment of dendritic cells to synovial fluid and tissues in the joint in RA. These dendritic cells are responsible for the secretion of chemokines such as CCL3, CCL17, CXCL19 and CXCL10 which leads to the influx of proinflammatory immune cells and recruitment of T cells to the RA synovium (Moret et al., 2013; Yu and Langridge, 2017). Again, in human RA patients, CD4 + CD25 + Foxp3+ Treg cells with high plasticity for the production of IL-17 has been documented to accumulate in the synovium (Möttönen et al., 2005; Komatsu et al., 2014). Other filamentous bacteria have demonstrated a robust ability to incite the gastrointestinal and systemic follicular helper T cell responses leading to massive production of autoantibodies in K/BxN mice (Teng et al., 2016).

The innate immune system relies mainly on receptors such as pattern recognition receptors (PRRs) which include Toll-like receptors (TLRs) and nucleotide-binding oligomerization domain-like receptors (NLRs). The interaction of TLRs and pathogen-associated molecular patterns (PAMPs) on microbes leads to a cascade of reactions leading to the initiation of inflammatory responses and the production of proinflammatory cytokines such as interleukin-6, TNF-α, or interleukin-1β to eliminate the pathogen (Bennike et al., 2017). Individual species of microbes in the microbiome of the gut stimulate the innate immune system differently which ultimately affects the release of either pro-inflammatory or anti-inflammatory cytokines (Horta-Baas et al., 2017). The upregulation of Th17 differentiation tied to the suppression of Treg cells leads to a shift in homeostasis towards inflammation (Horta-Baas et al., 2017).

Gut microbiome alterations may potentiate an increase in the permeability of the epithelia in the gut. These changes allow microbes and their products to cross into the lamina propria and sub-epithelial spaces. RA, like many other autoimmune diseases is preceded by autoimmunity marked by the presence autoantibodies. However, not all patients with these autoantibodies develop into RA and even in some patients there is seroreversion from positive to negative. This implies that the development of autoantibodies is reversible and requires additional triggers to transition into a chronic autoimmune state that eventually leads to RA. This transition is believed to be mediated by HLA class II with T cells playing a crucial role. Again, before the onset of arthritis in experimental animals, Th17 cells aggregate in germinal centers thereby suppressing the expression of sialyltransferases in plasmablasts. This causes the expression of IgG molecules with Fc-glycans that lack sialic acid thereby affecting the activity of the antibody (Nimmerjahn et al., 2007; Raju, 2008; Hayes et al., 2014). These interactions although poorly understood are believed to the associated the production of autoantibodies by pro-inflammatory T cells such as Th1 and Th17 cells (Zhong et al., 2018). The release of these autoantibodies together with other cytokines into circulation carries them to tissues and organs leading to the activation of macrophages culminating in the release of pro-inflammatory cytokines such as IL-6, IL-1, TNF-α, and IL-17. On the other hand, some microbes within the gut microbiome play a protective role in RA. Thus, enhance the polarization of regulatory T cells (Tregs) leading to the activation of an anti-inflammatory pathways by the production of cytokines such as transforming growth factor-β (TGF-β), IL-4 and IL-10 (Zhong et al., 2018).

Again, protein molecules from the gut microbiome such as filamin A (FLNA) and N-Acetyl-glucosamine-6-sulfatase (GNS) can potentiate and induce autoimmunity in RA through molecular mimicry. This stems from the high degree of epitope homology between HLA-DR-presented GNS peptide and sulfatase proteins of *Parabacteroides* sp. and *Prevotella* sp. In a similar manner, the HLA-DR-presented FLNA peptide is also homologous with epitopes from proteins of the *Butyricimonas* sp. and *Prevotella* sp. (Pianta et al., 2017). This observed homology presents some form of cross-reactivity of T cell epitopes of some bacteria with self-epitopes. Other mechanisms implicating gut microbiota with RA is the increased permeability of the gut. This is buttressed by the research work of Chen et al. (2016) who demonstrated an increase in the permeability of the gut through the downregulation of the expression of tight junction proteins due to bacteria such as *Collinsella* sp. These observations can partly be attributed to the changes in the redox environment, transport and metabolism of molecules such as zinc, iron and sulphur (X. Zhang et al., 2015).

**Figure 1:**
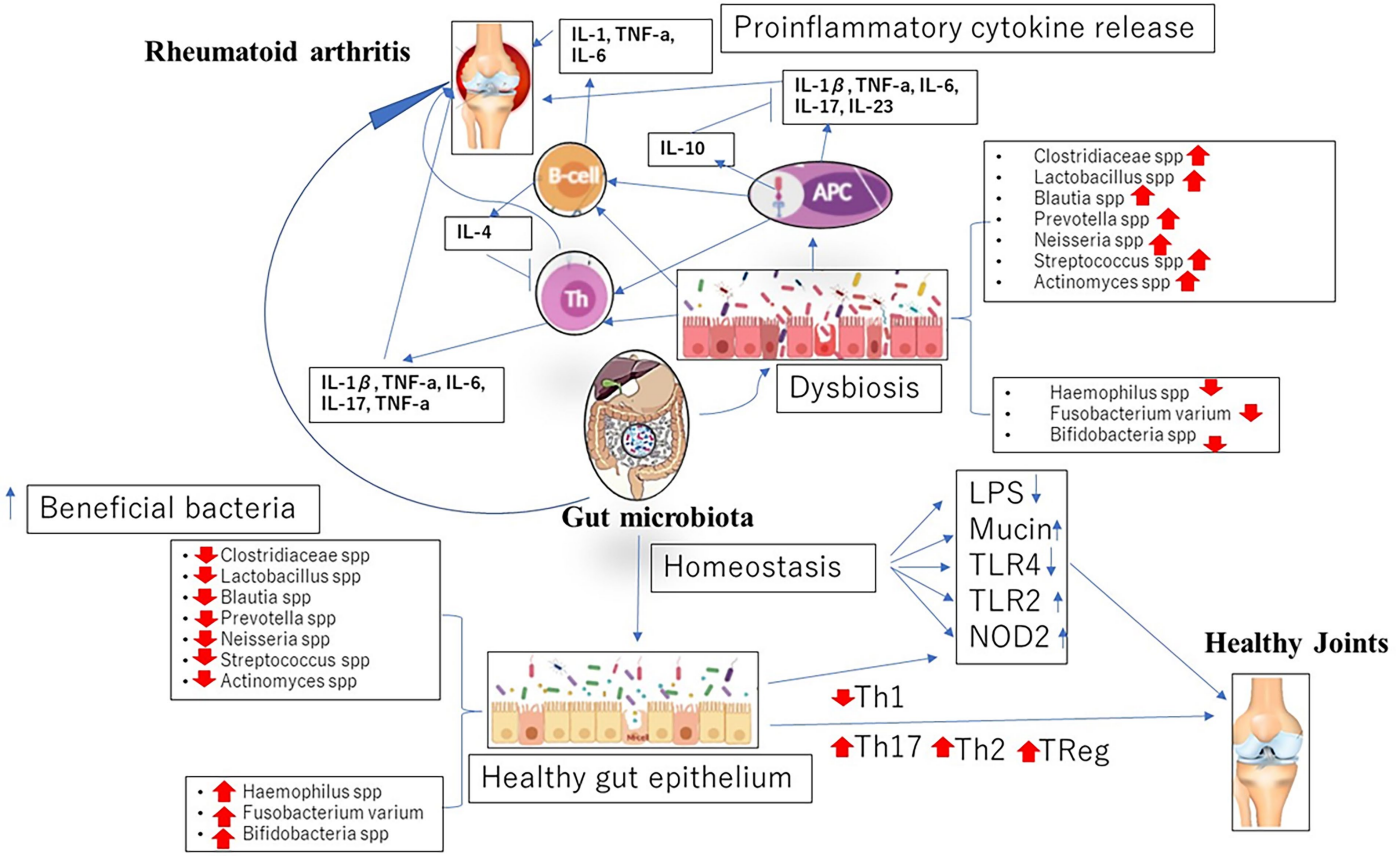
Dysbiosis in gut microbiome associated with the initiation and progression of rheumatoid arthritis (RA).

Aside these mechanisms, current studies have suggested enzymatic initiation of peptide citrullination, antigenic mimicry and stimulation of APCs by influencing TLRs or NOD-like receptors (NLRs) as other means by which the gut microbiome lead to the initiation of RA (Li and Wang, 2021; Figure 1).

### Probiotic effect on microbiome and RA

Probiotics refer to a range of live microbes that are administered in acceptable amounts to provide essential health benefits to the host (Hill et al., 2014). Moreover, myriads of studies have opined on the use of probiotics as a prospective adjuvant for the therapeutic management of RA (Ciccia et al., 2016; Wang et al., 2016; Reyes-Castillo et al., 2021). Although the mechanisms behind these observations remain partly unclear, it is believed that exogenous bacteria exert a temporary effect on the gut microbiome causing alterations to correct and improve dysbiosis-related diseases of which RA is one (Zhang et al., 2016). This effect is tied to the correction of imbalance in the cytokine levels in RA patients (Mohammed et al., 2017). The upregulation of pro-inflammatory cytokines coupled with a decrease in anti-inflammatory cytokines is a determining factor for the initiation and progression of RA. However, studies have documented in RA patients a reduction in the levels of IL-6, a key cytokine associated with the progression and severity of RA after being on probiotics (Brzustewicz and Bryl, 2015).

Probiotics-rich diet has been reported to ameliorate some of the symptoms of RA and this observation has been linked to the restoration of barrier mechanisms in the gut mucosal due to dysregulation (Mohammed et al., 2017). These barriers are restored by maintaining a healthy balance between gut microflora whiles resisting the colonization and translocation of harmful microbes. Again, barrier mechanisms are further enhanced by probiotics through the stimulation of mucus secretion from intestinal epithelial cells (Liu et al., 2016).

Again, probiotics have proven to directly modulate the immune system (La Fata et al., 2018; Kalinkovich and Livshits, 2019). Here, they have been reported to reduce inflammation by significantly downregulating the expression of TLRs (Gómez-Llorente et al., 2010) which are key components in the activation of signaling pathways leading to the production of cytokines for immune modulation (Cristofori et al., 2021). Furthermore, not only TLRs have been implicated to be stimulated by probiotics to produce cytokines, antigen presenting cells (APCs) have also been reported (Azad et al., 2018). In a related study, Kwon et al. (2010) also have reported on the induction of Treg immune response in experimental models of RA by probiotics. This was evident in the conversion of T cells into Tregs bearing FOXP_3_ which can regulate and suppress inflammatory cascade (Kwon et al., 2010). To our dismay, latest studies have collaborated with these earlier findings as probiotics have been implicated in the modulation of tryptophan metabolism and adenosine signaling in some experimental models. Here, specific probiotic strains have been noted for the activation of adenosine receptors which suppress the effect of Tregs on effectors T cells (He et al., 2017; Gao et al., 2018).

**Figure 2:**
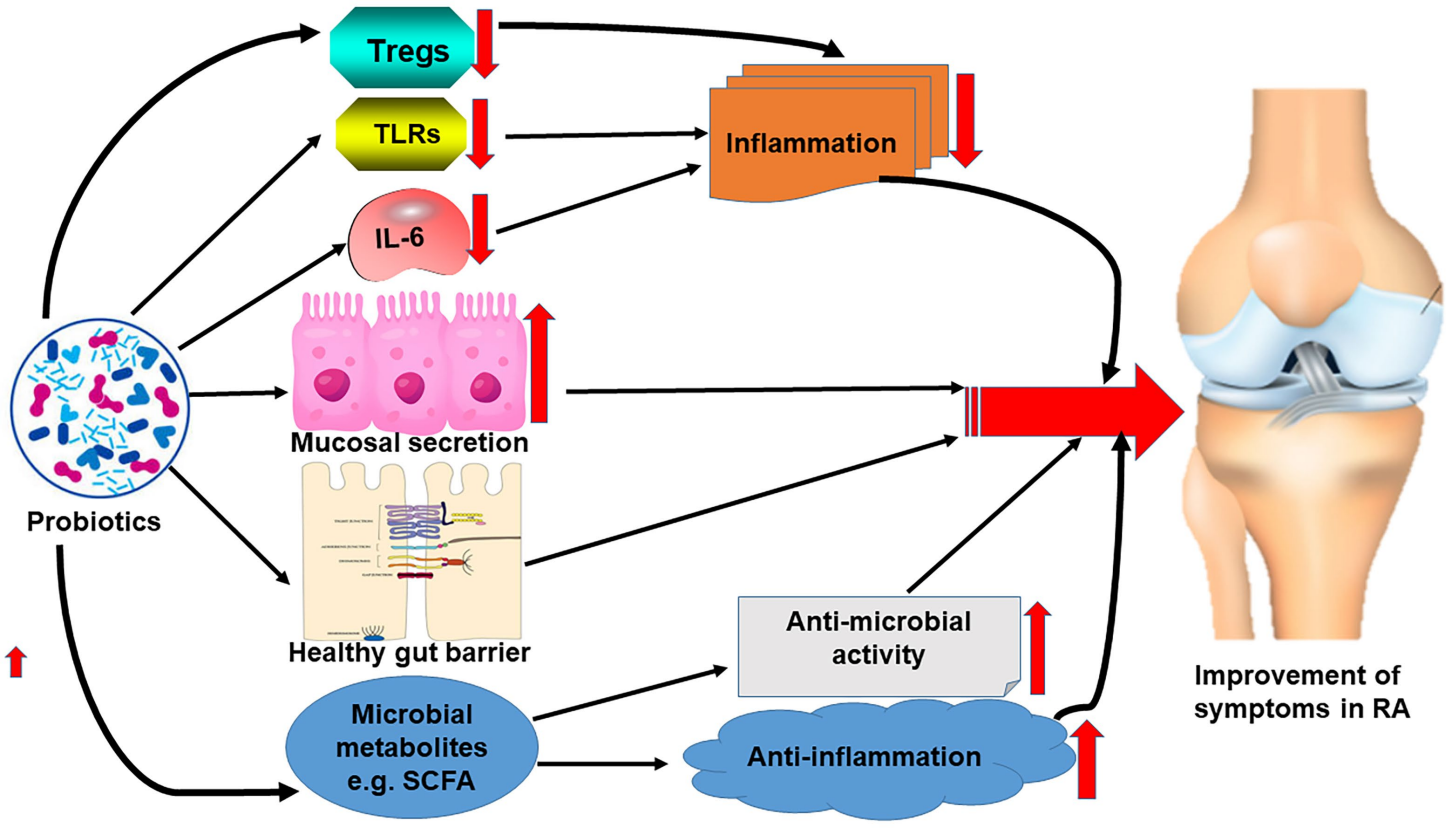
Effect of probiotics on the correction of gut microbiome dysbiosis leading to the reduction of the symptoms of RA.

Interestingly, metabolites such as short-chain fatty acids of the gut microbiome established due to the effect of probiotics reportedly have both antimicrobial and anti-inflammatory effects contributing to the alleviation of some symptoms of RA as observed in myriads of studies (Pirozzi et al., 2018; Lee et al., 2019; Figure 2).

## Conclusion

The alarming incidence of RA coupled with the difficulties associated with its management is a worrying challenge to stakeholders. With the implication of gut microbiome dysbiosis at the core of RA, supplements and adjuvants that can correct these alterations will be more than welcome. With a plethora of studies suggestive of the protective and corrective role of probiotics in the dysbiosis associated with RA, more research will be needed for its prospective therapeutic application. Again, probiotic strains with this beneficial role will have to be studied extensively to ensure their lack of pathogenicity.

## Author contributions

YO developed the idea. YO, KA, GG-Q, JA, FB-E, EO, CK, and RK-M wrote and reviewed the manuscript. YO and KA designed the figures. All authors read and approved the final version of the manuscript.

## Conflict of interest

The authors declare that the research was conducted in the absence of any commercial or financial relationships that could be construed as a potential conflict of interest.

## Publisher’s note

All claims expressed in this article are solely those of the authors and do not necessarily represent those of their affiliated organizations, or those of the publisher, the editors and the reviewers. Any product that may be evaluated in this article, or claim that may be made by its manufacturer, is not guaranteed or endorsed by the publisher.
